# From Vulnerability to Action: Advancing Health Equity for Rural and Underserved Populations

**DOI:** 10.3390/healthcare14213597

**Published:** 2026-10-23

**Authors:** Xiang-Yu Hou, Lijun Fan, Chiung-Jung (Jo) Wu

**Affiliations:** 1 Thompson Institute, University of the Sunshine Coast, Sippy Downs, QLD 4556, Australia; 2 School of Public Health, Southeast University, Nanjing 210009, China; 3 College of Health Sciences-Innovations in Health Sciences, VinUniversity, Hanoi 100000, Vietnam; 4 School of Health, University of the Sunshine Coast, Sippy Downs, QLD 4556, Australia; 5 Royal Brisbane and Women’s Hospital (RBWH), Herston, QLD 4006, Australia

## 1. Vulnerability, Wellbeing and Health Equity

Across countries and healthcare systems, patterns of health, illness and wellbeing continue to reflect differences in socioeconomic circumstances, geography, access to resources and healthcare, migration and cultural context, and the environments in which people are born, grow, live, work and age. The social determinants of health literature have consistently demonstrated that these conditions generate systematic differences in both exposure to health risks and opportunities to achieve good health [1,2]. More recent attention to structural determinants has further emphasised that such differences cannot be understood solely through individual characteristics or behaviours; they are shaped by the organisation of societies, institutions and health systems, including how resources, opportunities and power are distributed [3,4,5]. The language of “vulnerable populations” is commonly used to identify groups experiencing elevated health risks or barriers to healthcare, which can draw attention to populations whose needs may otherwise remain insufficiently recognised. However, it also carries a conceptual risk that vulnerability can inadvertently be treated as an inherent characteristic of particular groups rather than something that is created, intensified or mitigated through their social, economic, geographic and healthcare environments.

The papers published in this Special Issue, entitled *Research on Wellbeing and Health for Vulnerable Populations*, illustrate this complexity. They encompass healthcare deserts and avoidable mortality in Mexico [6], substance-use disorders in the Great Plains of the United States [7], mammography uptake among migrant women in Austria [8], neuropsychological development among socioeconomically disadvantaged children in Guatemala [9], and psychological distress following cardiac surgery [10] in Australia. At first glance, these populations, health problems and settings appear markedly different. Yet collectively, the papers point towards a common question: what creates or intensifies vulnerability, and what might be changed to improve people’s opportunities for health and wellbeing?

This Editorial considers the five contributions through that lens. We argue that recognising vulnerable populations is important, but insufficient. The next challenge is to identify vulnerability-producing conditions, determine which are modifiable, and translate knowledge about disparities into decisions and actions capable of reducing them.

## 2. What This Special Issue Contributes

### 2.1. Rural Place, Access and Healthcare Equity

Place matters for health, but geography does not operate independently of the systems built within it. It influences the healthcare workforce, infrastructure and services available, the distances people must travel, and the timeliness and continuity of care for them. International evidence demonstrates substantial geographic variation in healthcare access and quality, with potentially avoidable mortality providing an important indication of health-system performance [11,12,13,14,15].

The study by López-Yáñez et al. (2026) [6] provides a particularly striking illustration. Examining 1891 Mexican municipalities between 2015 and 2024, the authors found that 62.8% lacked hospital beds and were classified as healthcare deserts, and substantially higher avoidable mortality even after adjustment for social deprivation and other factors. Moreover, the disparity widened markedly following the COVID-19 pandemic. The study demonstrates that geographic disadvantage is not only a matter of physical distance, but also intersects with healthcare infrastructure and social deprivation, producing unequal opportunities to access care capable of preventing or treating premature mortality. This distinction matters. Describing a community as geographically disadvantaged can make the problem appear inevitable; identifying inadequate healthcare infrastructure as one contributor immediately introduces questions of resource allocation, service planning and policy.

Nahian and McFadden (2024) [7] similarly demonstrate the importance of geography in their examination of substance-use diagnoses before and during the COVID-19 pandemic in the US Great Plains. Their findings show demographic and geographic differences in patterns of diagnoses and remission in alcohol, opioid and stimulant use disorders, including differences between metropolitan and non-metropolitan populations. Such patterns need to be considered alongside longstanding evidence of rural barriers to substance-use treatment, including geographic disparities in access to opioid treatment programmes, medications and specialist addiction services [16,17,18].

Together, these contributions demonstrate that place can become a mechanism of vulnerability when healthcare resources and service models fail to compensate for geographic disadvantage. Rurality, remoteness or residence in an underserved municipality does not intrinsically determine poorer health. The consequences of place depend partly on decisions about where services, the workforce and other health-supporting resources are located.

### 2.2. Social Position, Migration and Unequal Opportunities for Health

Health disparities also emerge through differences in social and economic resources and people’s ability to access and benefit from preventive and healthcare services. Wahidie et al. (2024) [8] examined mammography participation among migrant and non-migrant women in Austria. Although mammography uptake tended to be lower across migrant groups, important differences existed between groups, with statistically significant disparities identified for women from particular countries of origin. Their findings demonstrate that we should not treat migrants as a homogeneous population and support diversity-sensitive approaches to healthcare.

This is consistent with the broader migration and health literature. Migration intersects with language, socioeconomic position, legal and administrative systems, health literacy, discrimination, cultural context and the organisation of healthcare itself [19,20]. Consequently, identifying lower screening participation among migrant populations should be the beginning of inquiry. The more actionable questions concern which barriers account for the difference and whether those barriers can be changed. Emerging evidence on interventions, such as language-concordant approaches to increasing cancer-screening participation, demonstrates how identification of a disparity can lead to testing practical responses [21].

Socioeconomic circumstances can influence health much earlier in the life course. Rodríguez-Prieto et al. (2024) [9] present a protocol for a randomised controlled trial of a neuropsychological stimulation programme for children from low socioeconomic backgrounds in Guatemala. The study responds to an extensive literature demonstrating associations between childhood poverty, cognitive and biological development and educational achievement [22,23]. Importantly, however, the proposed research does not stop at documenting disadvantage. It tests whether a structured intervention targeting cognitive and emotional development can improve academic performance and wellbeing.

The transition from observing unequal outcomes to testing whether they can be altered is particularly important. Socioeconomic disadvantage is often treated as immutable, which risks converting social explanation into therapeutic pessimism. Research capable of identifying modifiable pathways and testing interventions offers a route from understanding disadvantage towards improving opportunity.

### 2.3. Psychosocial Vulnerability Within Healthcare

The contribution by McCann et al. (2023) [10] broadens the concept of vulnerability further. Their review demonstrates that vulnerability can exist even when access to sophisticated healthcare has already been achieved. Psychological distress following cardiac surgery is common and can persist during recovery. The review identified personal, social and healthcare-related factors associated with postoperative distress, including social relationships, pain, functional impairment, pre-operative education and participation in cardiac rehabilitation. This evidence is important because healthcare success is frequently conceptualised through clinical endpoints, such as successful surgery, survival, complications, readmission or physiological recovery. Yet patients experience illness and recovery within psychological and social contexts. Previous research has similarly demonstrated relationships between psychosocial vulnerability and recovery following heart surgery [24], while depression remains clinically important in cardiovascular disease [25,26].

Thus, equitable healthcare cannot be reduced to providing equal access to a procedure. It also requires attention to people’s different capacities and resources to experience, recover from and ultimately benefit from healthcare. Across the five contributions, vulnerability therefore appears at different points along the health trajectory: before illness, through unequal social and developmental opportunities; in accessing preventive services and treatment; through the geographic distribution of healthcare resources; and during recovery after treatment. This diversity provides the basis for a broader interpretation of vulnerability.

## 3. From Vulnerable Populations to Vulnerability-Producing Conditions and Actions

A central lesson from this Special Issue is that vulnerability should not simply be treated as a characteristic of particular populations. It can also be understood as an outcome of the conditions in which people live and the systems with which they interact. This distinction is more than semantic.

Labelling a population as vulnerable directs attention towards who experiences disadvantage. Examining vulnerability-producing conditions asks why disadvantage occurs, through which mechanisms, and where intervention is possible. The five papers illustrate that vulnerability emerges from an interaction of people, place, resources and systems: Geographic isolation becomes consequential when healthcare infrastructure and workforce capacity and service models are insufficient to compensate for distance and limited local resources; migrant status may intersect with service accessibility; socioeconomic disadvantage can shape children’s developmental opportunities through educational and psychosocial pathways; substance-use outcomes are influenced by treatment availability; psychological distress following cardiac surgery can be affected by social support and education.

The distinction also has implications for health equity. Equal treatment is not necessarily equitable treatment when people face unequal barriers and possess unequal resources with which to benefit from healthcare. The principle of proportionate universalism, for example, proposes that healthcare actions should be universal but implemented at a scale and intensity proportionate to the level of disadvantage [27]. More broadly, achieving health equity may require greater investment in populations experiencing greater barriers or needs [28].

The more difficult task in health equity is to make equity visible in what we measure, whom we include, how resources are allocated, how services are designed, what health professionals are taught, and which research questions we choose to pursue.

For researchers: Descriptive research remains necessary. Without measurement, inequities can remain invisible. In addition to demonstrating that one population experiences poorer outcomes than another, research should examine mechanisms and modifiable determinants, investigate heterogeneity within populations, and evaluate interventions designed to reduce disparities.

Evaluation should also ask whether interventions affect distribution as well as averages. An intervention that improves overall population health while disproportionately benefiting already advantaged populations may inadvertently widen disparities.

For policymakers and funders: Commitment to health equity has limited meaning if resource-allocation systems remain insensitive to differences in need, cost and barriers to access. The healthcare-desert findings in this Special Issue make this particularly tangible [6]. If inadequate infrastructure contributes to avoidable mortality, the response cannot rest solely with individuals or clinicians. It becomes a question of system design and allocation.

Equity may therefore require unequal inputs to achieve fairer opportunities for outcomes. Funding formulas, workforce policies, infrastructure investment and programme design should explicitly consider geography, socioeconomic disadvantage, population need and barriers to accessing care. Importantly, equity objectives should be accompanied by measurable indicators so that governments and organisations can determine whether investments are actually narrowing disparities.

For healthcare organisations and practitioners: Offering the same service to everyone does not mean everyone has the same opportunity to use or benefit from it. A screening programme may technically be available to migrant and non-migrant women alike, yet language, culture, and service design may produce very different levels of participation. Healthcare organisations should therefore examine pathways from the patient’s perspective.

For educators: Health-professional education often introduces learners to populations described as vulnerable or underserved. While awareness is important, lists of vulnerable groups can unintentionally reinforce the idea that vulnerability resides within people themselves.

Education should instead help future health professionals understand the pathways through which social conditions, geography, policy, resource allocation and healthcare structures influence individual health. Health students should be equipped to see the patient within a broader context, from the person, through the family, community and place to the system, rather than diagnosis alone. Such education also encourages reflexivity about the fact that healthcare professionals and institutions themselves participate in systems that can reduce, reproduce or amplify disadvantage.

Therefore, in summary, our challenge is not simply to increase attention to vulnerable populations; it is to identify and reduce the conditions that produce or intensify vulnerability. Health equity ultimately becomes meaningful when it is reflected in what we do, how we measure inequities, allocate resources, design services, educate health professionals, evaluate interventions and make equity-based decisions.
